# The burden of Neglected Tropical Diseases in Brazil, 1990-2016: A subnational analysis from the Global Burden of Disease Study 2016

**DOI:** 10.1371/journal.pntd.0006559

**Published:** 2018-06-04

**Authors:** Francisco Rogerlândio Martins-Melo, Mariângela Carneiro, Alberto Novaes Ramos, Jorg Heukelbach, Antonio Luiz Pinho Ribeiro, Guilherme Loureiro Werneck

**Affiliations:** 1 Federal Institute of Education, Science and Technology of Ceará, Caucaia, Ceará, Brazil; 2 Institute of Studies in Public Health, Federal University of Rio de Janeiro, Rio de Janeiro, Rio de Janeiro, Brazil; 3 Departamento de Parasitologia, Instituto de Ciências Biológicas, Universidade Federal de Minas Gerais; 4 Department of Community Health, School of Medicine, Federal University of Ceará, Fortaleza, Ceará, Brazil; 5 Hospital das Clínicas and School of Medicine, Universidade Federal de Minas Gerais, Belo Horizonte, Minas Gerais, Brazil; 6 Department of Epidemiology, Social Medicine Institute, State University of Rio de Janeiro, Rio de Janeiro, Rio de Janeiro, Brazil; Sacro Cuore Hospital, ITALY

## Abstract

**Background:**

Neglected Tropical Diseases (NTDs) are important causes of morbidity, disability, and mortality among poor and vulnerable populations in several countries worldwide, including Brazil. We present the burden of NTDs in Brazil from 1990 to 2016 based on findings from the Global Burden of Diseases, Injuries, and Risk Factors Study 2016 (GBD 2016).

**Methodology:**

We extracted data from GBD 2016 to assess years of life lost (YLLs), years lived with disability (YLDs), and disability-adjusted life-years (DALYs) for NTDs by sex, age group, causes, and Brazilian states, from 1990 to 2016. We included all NTDs that were part of the priority list of the World Health Organization (WHO) in 2016 and that are endemic/autochthonous in Brazil. YLDs were calculated by multiplying the prevalence of sequelae multiplied by its disability weight. YLLs were estimated by multiplying each death by the reference life expectancy at each age. DALYs were computed as the sum of YLDs and YLLs.

**Principal findings:**

In 2016, there were 475,410 DALYs (95% uncertainty interval [UI]: 337,334–679,482; age-standardized rate of 232.0 DALYs/100,000 population) from the 12 selected NTDs, accounting for 0.8% of national all-cause DALYs. Chagas disease was the leading cause of DALYs among all NTDs, followed by schistosomiasis and dengue. The sex-age-specific NTD burden was higher among males and in the youngest and eldest (children <1 year and those aged ≥70 years). The highest age-standardized DALY rates due to all NTDs combined at the state level were observed in Goiás (614.4 DALYs/100,000), Minas Gerais (433.7 DALYs/100,000), and Distrito Federal (430.0 DALYs/100,000). Between 1990 and 2016, the national age-standardized DALY rates from all NTDs decreased by 45.7%, with different patterns among NTD causes and Brazilian states. Most NTDs decreased in the period, with more pronounced reduction in DALY rates for onchocerciasis, lymphatic filariasis, and rabies. By contrast, age-standardized DALY rates due to dengue, visceral leishmaniasis, and trichuriasis increased substantially. Age-standardized DALY rates decreased for most Brazilian states, increasing only in the states of Amapá, Ceará, Rio Grande do Norte, and Sergipe.

**Conclusions/Significance:**

GBD 2016 findings show that, despite the reduction in disease burden, NTDs are still important and preventable causes of disability and premature death in Brazil. The data call for renewed and comprehensive efforts to control and prevent the NTD burden in Brazil through evidence-informed and efficient and affordable interventions. Multi-sectoral and integrated control and surveillance measures should be prioritized, considering the population groups and geographic areas with the greatest morbidity, disability, and most premature deaths due to NTDs in the country.

## Author summary

Neglected Tropical Diseases (NTDs) are a public health problem in Brazil. We used findings from the Global Burden of Disease Study 2016 (GBD 2016) to explore the burden of NTDs in Brazil by sex, age group, specific causes, and Brazilian states from 1990 to 2016. In 2016, NTDs caused 475,410 disability-adjusted life-years (DALYs) (95% uncertainly interval [UI]: 337,334–679,482; equating to an age-standardized rate of 232.0 DALYs/100,000 population) in Brazil. Chagas disease was the main cause of DALYs among all NTDs. The disease burden was higher among males, in the youngest and eldest (children under 1 year and elderly aged 70 years and older), and in Brazilian states considered endemic for the major NTDs. There was a consistent reduction in overall age-standardized DALY rates for all NTDs combined (overall reduction of 45.7%) and most NTD causes between 1990 and 2016, but with a pronounced increase for dengue and visceral leishmaniasis. Despite of the remarkable progress in reducing the DALY rates, NTDs remain as important preventable and neglected causes of disability and premature death in Brazil. GBD 2016 results call for intensified and comprehensive efforts to prevent and reduce the burden of NTDs in Brazil, with special emphasis on less developed areas and vulnerable populations.

## Introduction

Neglected Tropical Diseases (NTDs) are a group of communicable diseases that affect predominantly the poor and vulnerable populations in about 150 countries mainly in Africa, Asia, and Latin America and the Caribbean [1]. Most NTDs are stigmatizing, disabling, debilitating, and cause poverty-promoting chronic conditions and preventable causes of death [1,2]. NTDs affect more than 1.5 billion people, and about 3 billion people are at risk to acquire one or more NTDs worldwide [2–4]. About 150,000–500,000 deaths are attributed to NTDs annually [5,6]. Despite being endemic mainly in low and middle income countries, their occurrence has been on the rise in high income countries, due to the increasing population mobility and migratory movements worldwide in the past decades [2,7].

In Brazil, most of the world’s important NTDs are present, being responsible for the majority of the burden in Latin America [8–10]. The country recorded the largest number of cases of leprosy, dengue, schistosomiasis, Chagas disease, and leishmaniases in the region [9]. NTD burden varies by Brazilian regions, with most of these diseases occurring in areas of low socioeconomic status, mainly in the North and Northeast regions [8]. About 8,000–10,000 NTD-related deaths are recorded in Brazil annually, mostly for Chagas disease [10,11]. However, the true NTD burden is considered to be underestimated in Brazil [8,10].

The Global Burden of Diseases, Injuries, and Risk Factors Study (GBD) is a comprehensive and updated worldwide epidemiological study that aims at quantifying the mortality, morbidity, and disability of major diseases, injuries, and risk factors by location, sex, age group, and time period [12]. GBD study uses as the main population health metric the disability-adjusted life years (DALYs), a measure of health loss due to both fatal and non-fatal disease burden [12]. DALYs are estimated by summing years lived with disability (YLDs) and years of life lost (YLLs) due to premature mortality for a given cause [12–14]. GBD 2016 estimated a total of 15 million DALYs due to NTDs worldwide in 2016 (sum of DALYs from the 15 of 18 NTDs appearing in the priority list of the World Health Organization [WHO]) [12]. Soil-transmitted helminthiases (STHs) (3.3 million DALYs), dengue (3.0 million DALYs), and schistosomiasis (1.9 million DALYs) were the main causes of burden among all NTDs in 2016 [12]. Sub-Saharan Africa (5.3 million DALYs) and South Asia (4.1 million DALYs) were the regions with the highest NTD burden [12].

Despite their health, economic and social impact, few systematic and comprehensive studies to quantify and compare the disease burden due to NTDs have been conducted in Brazil to date. The quantitative assessment and timely information of NTD burden in endemic areas are important to guide health policy, allocate resources appropriately, measure progress, and monitor the effectiveness and impact of health interventions and for surveillance, prevention, and disease control programs [10,11,15,16]. Using GBD 2016 data, we assessed the burden of NTDs in Brazil by sex, age group, and Brazilian states, from 1990 to 2016.

## Methods

### Study area

Brazil, officially called the Federal Republic of Brazil, is South America’s largest country and has a total territory of 8.5 million km^2^ with an estimated population of 207.7 million inhabitants in 2017 [17]. The country is divided politically and administratively into 27 federative units (26 states and the Federal District) and 5,570 municipalities, grouped into five geographic macro-regions (South, Southeast, Central-West, North, and Northeast). Brazil has the highest gross domestic product (GDP) among Latin America countries and the ninth of the world in 2016 [18], but remains a country with a high income inequality (Gini index of 0,549 in 2017) [19]. Despite the considerable reduction of poverty until 2014, about 12.1% of the Brazilian population were living in extreme poverty in 2016 (proportion of people with monthly household income per capita of up to ¼ of the minimum wage), and with remarkable variations regions: 23.1% in Northeast, 22.7% in North, 6.3% in Southeast, 6.0% in Central-West, and 4.7% in South region [20].

### GBD overview

This research has been conducted as part of the GBD study, coordinated by the Institute for Health Metrics and Evaluation (IHME) at the University of Washington. Data from the GBD 2016 study were used to explore the burden of NTDs in Brazil from 1990 to 2016. Detailed description of methods and approach used in the GBD 2016 and for estimation of specific NTDs has been published elsewhere [12–14,21, 22]. Briefly, GBD 2016 provides a comprehensive annual assessment of mortality and morbidity estimates for 333 diseases and injuries and 84 risk factors for 195 countries and territories from 1990 to 2016 [12–14,22].

### NTD case definition

The GBD 2016 cause list hierarchy is organized into four levels of causes that are mutually exclusive and collectively exhaustive [12,14]. Level 1 has three broad categories: communicable, maternal, neonatal, and nutritional (CMNN) disorders; non-communicable diseases (NCDs); and injuries. Level 2 has 21 cause groups, such as neoplasms and cardiovascular diseases. Levels 3 and 4 are disaggregated in 168 and 276 causes, respectively [12].

NTD-related causes are included in the level 2 group “Neglected tropical diseases and malaria”, which consists of 20 infectious and parasitic diseases including malaria, NTDs prioritized by the WHO (15 of the 18 NTDs in 2016), and other neglected diseases such as yellow fever, Ebola virus disease, and Zika virus disease [12,14]. Most of the WHO’s priority NTDs are part of the GBD 2016 cause list, but Buruli ulcer, chikungunya virus disease (included as “other NTDs”), mycetoma, and yaws are not currently estimated and available by GBD study [12,14,16].

In this study, we included GBD 2016 estimates for 12 NTD causes that are part of the official WHO priority list in 2016 and that are endemic/autochthonous in Brazil [1,2,10,11]: Chagas disease, leishmaniases (visceral and cutaneous/mucocutaneous leishmaniasis), schistosomiasis, cysticercosis, cystic echinococcosis, lymphatic filariasis, onchocerciasis, trachoma, dengue, rabies, soil-transmitted helminthiases (STHs) (or intestinal nematode infections as designated in the GBD studies: ascariasis, trichuriasis, and hookworm disease), and leprosy. Although fascioliasis (one of the trematode worm infections included within the GBD category of food-borne trematodiases) is endemic in animals and humans in some areas of Brazil, especially in southern states [23,24], its burden was not estimated for the country in the GBD 2016.

NTD causes were defined and identified according to the International Classification of Diseases, 9th Revision (ICD-9) and 10th Revision (ICD-10) [13,14]. The ICD definitions and modeling strategy for each NTD cause used in this study are described in detail elsewhere [13,14].

### Geographic units and time period

In this study, we present NTD burden estimates at national (entire country) and subnational level (27 federative units [26 states and the Federal District], herein simply named as states).

GBD 2016 estimated cause-specific burden for the years 1990–2016 [12]. Here we focused on NTD burden estimates for 2016, with reference to changes in the burden since 1990. All GBD 2016 results and metrics by location and year are available at http://vizhub.healthdata.org/gbd-compare.

### Data sources

The GBD 2016 approach to estimate all-cause and cause-specific mortality has been described previously [13,21]. To assemble a comprehensive cause of death database, the GBD study uses all accessible data sources that meet quality criteria and rigorous analysis, and corrects for known bias in each data source [13]. Briefly, data sources included vital registration systems, verbal autopsy data, cancer registries, surveillance data for maternal mortality, injuries, and child death, census and survey data for maternal mortality and injuries, and police records for interpersonal violence and transport injuries [13,21]. For Brazil, the main source of mortality data used in GBD study was the Mortality Information System (*Sistema de Informações sobre Mortalidade–*SIM) database of the Brazilian Ministry of Health, adjusted by other national and international sources [25–28]. Data corrections were made for mortality sub-registration and redistribution of garbage codes for defined causes based on the GBD 2016 redistribution algorithms [13,29]. Garbage codes are the assignment of causes of death that could not or should not be classified as the underlying cause of death [13]. For GBD, each death is attributed to a single underlying cause–the cause that initiated the series of events leading to death–in accordance with ICD principles [12,13]. GBD 2016 used the Cause of Death Ensemble model (CODEm), negative binomial regression, and natural history models to estimate the number of deaths for NTD causes by location, age, sex, and year [13]. These modeling strategies for individual NTD cause of death data were described in detail elsewhere [13].

Detailed descriptions of the GBD 2016 modeling strategy for morbidity estimation and validation have been published elsewhere [14]. GBD study uses all available data that met a minimum standard of acceptable quality for each disease [14]. To measure non-fatal disease burden, GBD 2016 used epidemiological surveillance data, published and unpublished disease registries, and published scientific reports [14]. GBD 2016 used DisMod-MR 2.1, a Bayesian-regression analytic tool, to synthesize consistent estimates of prevalence and incidence of non-fatal outcomes by age, sex, year, and location using a wide range of updated and standardized analytical procedures [14]. Detailed nonfatal modeling methods are described in detail elsewhere [14]. In Brazil, the main sources of morbidity data used in GBD 2016 are the morbidity national databases of the Brazilian Ministry of Health such as the Notifiable Disease Information System (*Sistema de Informação de Agravos de Notificação–*SINAN), Hospital Information System of the Brazilian Unified Health System (*Sistema de Informações Hospitalares do Sistema Único de Saúde–*SIH/SUS), Outpatient Information System of the Unified Health System (*Sistema de Informações Ambulatoriais do SUS–*SIA/SUS); specific disease databases such as the Schistosomiasis Control Program Information System (*Sistema de Informação do Programa de Controle da Esquistossomose–*SISPCE); national demographic and health surveys; and published scientific literature of Brazilian population-based disease prevalence studies [25,26].

GBD data sources for Brazil have been described in detail previously [25–28]. The input data sources and publications for each NTD in Brazil used in GBD 2016 can be accessed at http://ghdx.healthdata.org/gbd-2016/data-input-sources.

### DALY calculation

DALYs were used as the measure of total burden. DALYs are estimated by the sum of the years of life lost (YLLs) due to premature mortality and years lived with disability (YLDs) for a given disease or injury [12]. One DALY represents one year of healthy life lost [12,30]. Detailed methods of DALY estimation have been described in previous GBD publications [12,30].

YLLs are calculated multiplying the number of deaths from NTDs in each age group by the standard life expectancy at each age group [12,13]. For GBD 2016, the standard life expectancy at birth is 86.6 years, based on the lowest observed death rates for each 5-year age group in populations greater than 5 million people in 2016 [13]. YLDs are estimated by multiplying the prevalence of each sequelae or combination of sequelae from NTDs, in each age group, sex, location, and year, by their disability weights [12,14]. Disability weights were derived from population-based surveys of the general public [12,31]. Disability weight reflects the severity of health loss associated with the respective NTD on a scale varying from 0 (perfect health) to 1 (equivalent to death) [14]. For some NTDs in which death is considered a rare event, mortality was assumed to be zero. Thus, for these causes, YLLs were not calculated and DALYs were equal to the YLDs [12,32,33].

We present results in absolute numbers and age-standardized rates (per 100,000 population) of DALYs from NTDs (individually and as a group) by sex, age group, year, and location, with their respective 95% uncertainly intervals (UIs). Age-standardized DALY rates (per 100,000 population) were computed using the GBD world population standard [13,14,21]. We report positive and negative percentage changes to show increasing and decreasing trends from 1990 to 2016, respectively. We also present the expected estimates of NTD burden produced by GBD 2016 based on Socio-Demographic Index (SDI), a composite indicator based on the geometric mean of three measures: lag-distributed income per capita, average educational attainment over aged 15 years and older, and total fertility rate [12,13]. Additional detail on SDI calculation and location-specific SDI values are available elsewhere [13].

### Ethics statement

This study was based on secondary databases which are publicly available, without identification of individual data. The GBD Brazil study was approved by the Ethical Review Board of the Federal University of Minas Gerais, Belo Horizonte, Brazil (Project CAAE n° 62803316.7.0000.5149).

## Results

### NTD burden

In 2016, the selected NTDs caused an estimate of 475,410 DALYs (95% UI: 337,334–679,482; age-standardized DALY rate of 231.98/100,000 population) in Brazil, accounting for 0.8% of all-cause DALYs (about 57.1 million DALYs).

Table 1 presents the national estimates for the number of DALYs and age-standardized DALY rates for the selected NTDs and the percentage change between 1990 and 2016, as well as the expected age-standardized DALY rates based on SDI in 2016. Chagas disease was the leading cause of DALYs among all NTDs in 2016 (141,640 DALYs [95 UI: 129,065–155,941], age-standardized DALY rate of 70.69/100,000 population [95% UI: 64.49–77.81]), followed by schistosomiasis (102,259 DALYs [95 UI: 59,767–176,124], age-standardized DALY rate of 46.92/100,000 population [95%UI: 27.54–80.71]), and dengue (92,538 DALYs [95% UI: 63,477–130,370], age-standardized DALY rate of 44.87/100,000 population [95% UI: 30.85–63.10]) (Table 1). Based exclusively on SDI (0.71 for Brazil), observed age-standardized DALY rates for most NTDs were higher than expected, with the largest observed-to-expected ratios verified for schistosomiasis (428.4), visceral leishmaniasis (284.5), and trachoma (81.3) (Table 1).

**Table 1:** Absolute number of DALYs and age-standardized DALY rates (per 100,000 population) for neglected tropical diseases in Brazil, with percentage change between 1990 and 2016, and expected age-standardized DALY rates for 2016.

NTD cause	Number of DALYs (95% UIs)			Age-standardized DALY rate (per 100,000) (95% UIs)			Expected DALY rate– 2016*	O/E ratio
	1990	2016	% Change 1990–2016	1990	2016	% Change 1990–2016		
Chagas disease	223,878.6 (209,371.8–238,590.7)	141,640.3 (129,065.0–155,941.4)	-36.7	233.18 (219.34–247.29)	70.69 (64.49–77.81)	-69.7	8.01	8.83
Leishmaniases	20,081.3 (12,471.2–31,566.8)	40,967.4 (24,577.7–65,628.0)	104.0	12.34 (7.99–18.81)	22.40 (13.35–36.26)	81.6	0.14	165.22
* Visceral leishmaniasis*	*17*,*168*.*3 (9*,*696*.*9*–*28*,*176*.*8)*	*37*,*776*.*7 (21*,*610*.*8*–*62*,*522*.*7)*	*120*.*0*	*10*.*09 (6*.*11*–*16*.*42*	*20*.*90 (11*.*84*–*34*.*83)*	*107*.*2*	*0*.*07*	*284*.*51*
* Cutaneous and mucocutaneous leishmaniasis*	*2*,*913*.*0 (914*.*7*–*6*,*569*.*8)*	*31*,*90*.*7 (1*,*974*.*3*–*5*,*049*.*4)*	*9*.*5*	*2*.*25 (0*.*68*–*4*.*97)*	*1*.*50 (0*.*92*–*2*.*38)*	*-33*.*3*	*0*.*06*	*24*.*14*
Schistosomiasis	98,564.1 (61,743.0–162,482.0)	102,259.3 (59,766.7–176,123.6)	3.8	86.91 (55.21–141.88)	46.92 (27.54–80.71)	-46.0	0.11	428.36
Cysticercosis	26,627.2 (20,863.1–33,036.8)	15,142.9 (10,960.0–20,859.1)	-43.1	21.56 (16.72–27.08)	7.00 (5.08–9.62)	-67.5	14.31	0.49
Cystic echinococcosis	1,957.8 (1,379.4–3,065.1)	802.8 (553.4–1,307.0)	-59.0	1.45 (1.02–2.19)	0.38 (0.26–0.60)	-74.0	0.42	0.9
Lymphatic filariasis	201.8 (37.3–610.8)	9.9 (1.9–29.3)	-95.1	0.15 (0.03–0.46)	0.00 (0.00–0.01)	-97.0	0.02	0.22
Onchocerciasis	1,424.3 (947.9–1,975.0)	17.4 (2.1–63.8)	-98.8	1.00 (0.67–1.38)	0.01 (0.00–0.03)	-99.2	0.13	0.06
Trachoma	7,683.6 (5,267.5–10,815.2)	5,783.2 (3,860.4–8,313.3)	-24.7	12.88 (8.84–17.91)	3.43 (2.28–4.90)	-73.3	0.04	81.32
Dengue	1,698.6 (321.7–3,609.2)	92,538.4 (63,477.0–130,370.3)	5,347.8	1.09 (0.19–2.34)	44.87 (30.85–63.10)	4,015.5	1.11	40.42
Rabies	6,481.5 (4,582.2–9,791.7)	86.1 (74.0–103.4)	-98.7	3.41 (2.46–4.97)	0.04 (0.04–0.05)	-98.8	0.10	0.44
Intestinal nematode infections/Soil-transmitted helminths	88,020.2 (52,106.7–146,586.1)	72,101.9 (42,257.2–115,120.6)	-18.1	51.33 (30.78–85.14)	34.22 (20.18–54.59)	-33.3	15.34	2.23
* Ascariasis*	*56*,*674*.*9 (33*,*595*.*0*–*96*,*995*.*9)*	*24*,*218*.*9 (14*,*038*.*3*–*39*,*902*.*8)*	*-57*.*3*	*30*.*36 (17*.*96*–*51*.*73)*	*11*.*62 (6*.*83*–*19*.*08)*	*-61*.*7*	*3*.*46*	*3*.*36*
* Trichuriasis*	*2138*.*7 (1*,*112*.*8*–*3*,*781*.*2)*	*9*,*413*.*8 (5*,*030*.*0*–*16*,*084*.*8)*	*340*.*2*	*1*.*49 (0*.*77*–*2*.*64)*	*4*.*48 (2*.*40*–*7*.*57)*	*201*.*1*	*0*.*93*	*4*.*81*
* Hookworm disease*	*29*,*206*.*6 (17*,*485*.*6*–*46*,*922*.*2)*	*38*,*469*.*2 (22*,*153*.*8*–*61*,*581*.*3)*	*31*.*7*	*19*.*48 (11*.*59*–*31*.*50)*	*18*.*13 (10*.*45*–*29*.*01)*	*-7*.*0*	*10*.*96*	*1*.*66*
Leprosy	2,066.6 (1,388.2–2,899.7)	4,060.3 (2,738.6–5,621.9)	96.5	2.21 (1.50–3.09)	2.02 (1.36–2.79)	-8.8	0.30	6.61
**Total all NTDs**	**478,685.6 (370,479.9–645,029.2)**	**475,410.0 (337,333.9–679,481.6)**	**-0.7**	**427.51 (344.7–552.54)**	**231.98 (165.43–330.47)**	**-45.7**	**55.51**	**4.18**

NTDs = neglected tropical diseases. DALYs = disability-adjusted life-years. 95% UIs = 95% uncertainty intervals. O/E ratio = observed/expected DALY ratio.

*Expected age-standardized DALY rates (per 100,000 population) based on country’s Socio-demographic Index (SDI).

### Proportional contribution of DALY components

**Fig 1:**
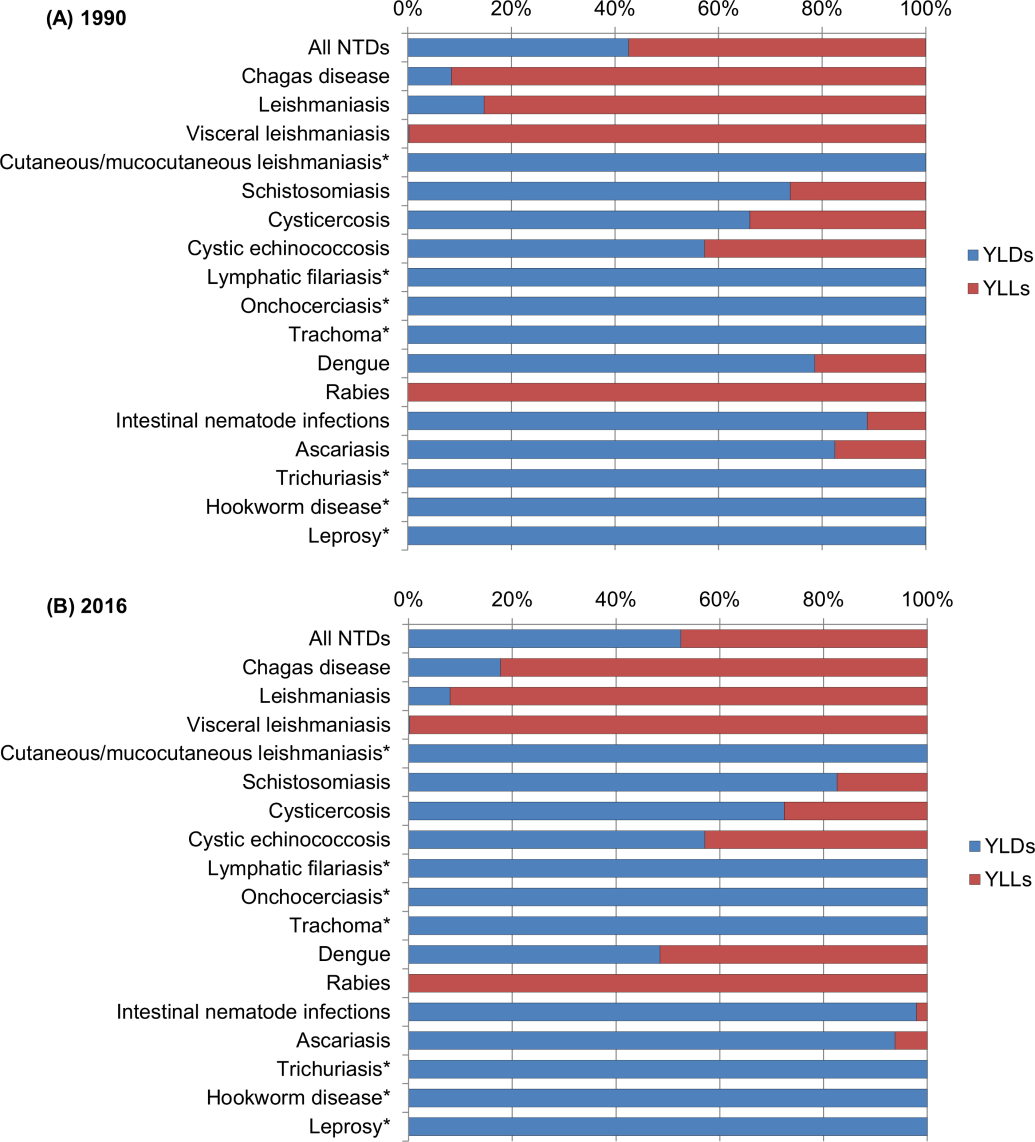
Contribution of YLDs and YLLs to total DALYs for each Neglected Tropical Disease in Brazil for (A) 1990 and (B) 2016. *For these causes, YLL is assumed to be zero. YLDs were equivalent to DALYs. YLDs = years lived with disability; YLLs = years of life lost; DALYs = disability-adjusted life-years; NTDs = neglected tropical diseases.

**Fig 2:**
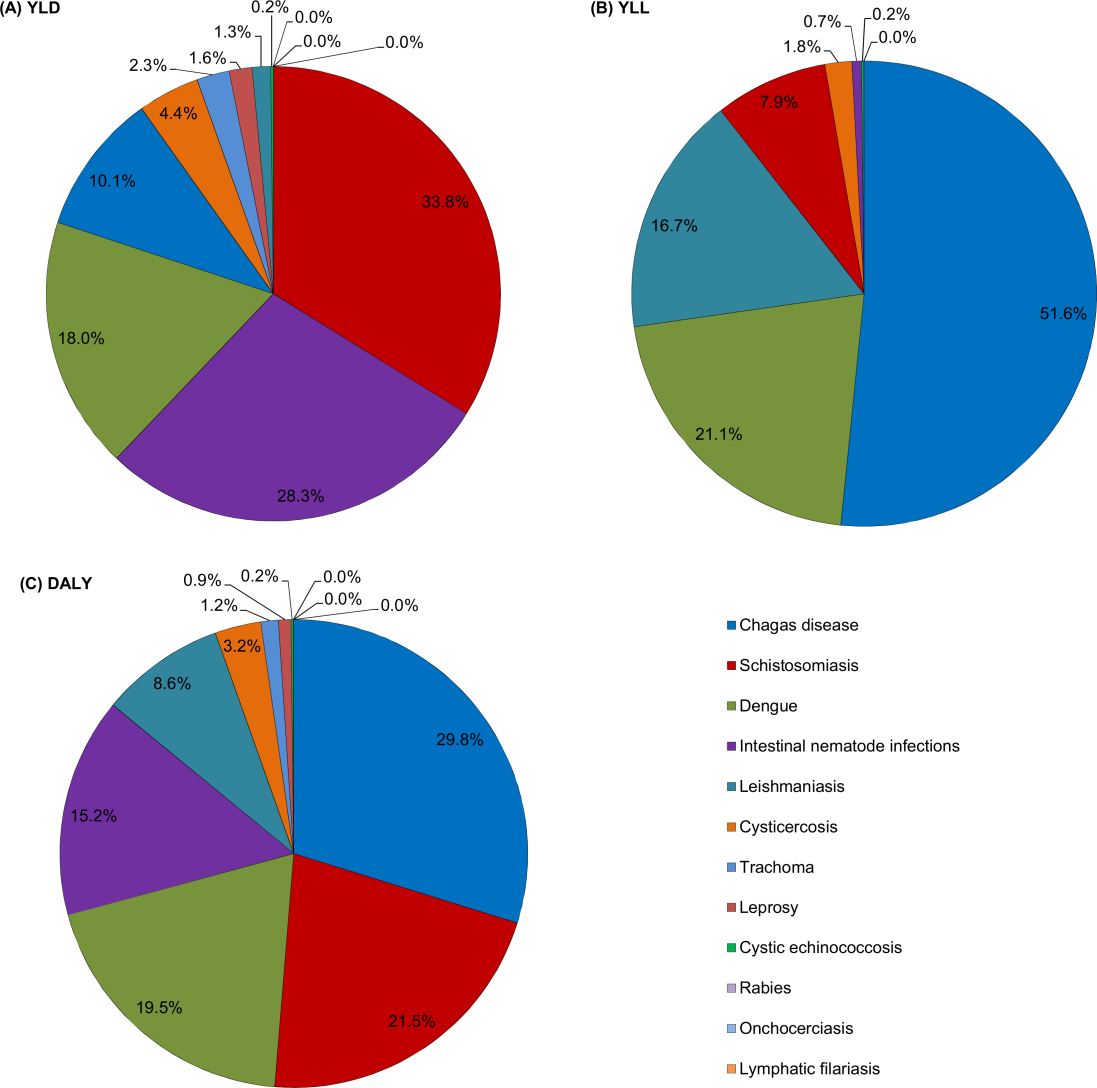
Proportion of (A) YLDs, (B) YLLs and DALYs (C) for each cause in relation to all NTDs combined in Brazil, 2016. YLDs = years lived with disability; YLLs = years of life lost; DALYs = disability-adjusted life-years; NTDs = neglected tropical diseases.

Fig 1 shows the contribution of YLDs and YLLs to total DALYs for each NTD in 2016, and Fig 2 illustrates the proportion of DALYs, YLDs e YLLs for each disease in relation to all NTDs. In 2016, most DALYs due to all NTDs combined were the result of the YLD component (52.5%; 249,636 YLDs vs. 225,774 YLLs) (Fig 1), while YLLs were the main component of DALYs for all NTDs in 1990 (57.4%; 274,605 YLLs vs. 204,081 YLDs). The proportion of YLLs and YLDs within DALYs varied by NTD in 2016. YLDs were the main component of DALYs for most NTDs, accounting for all DALYs for leprosy, lymphatic filariasis, onchocerciasis, trachoma, cutaneous/mucocutaneous leishmaniasis, hookworm, and trichuriasis, and contributed to more than 80% of DALYs for schistosomiasis and ascariasis (Fig 1). YLLs were the main component of DALYs for rabies (100.0%), visceral leishmaniasis (99.8%), Chagas disease (82.3%), and dengue (51.5%) (Fig 1).

Among all NTDs analyzed, Chagas disease accounted for the highest proportion of total DALYs (29.8% of DALYs for all NTDs combined), followed by schistosomiasis (21.5%), dengue (19.5%), and STHs (15.2%) (Fig 2). Schistosomiasis contributed for most of YLDs (33.8%) among all NTDs, followed by STHs (28.3%), and dengue (18.0), while Chagas disease accounted for 51.6% of YLLs, followed by dengue (21.1%) and visceral leishmaniasis (16.7%) (Fig 2).

### Changes in NTD burden over time

**Fig 3:**
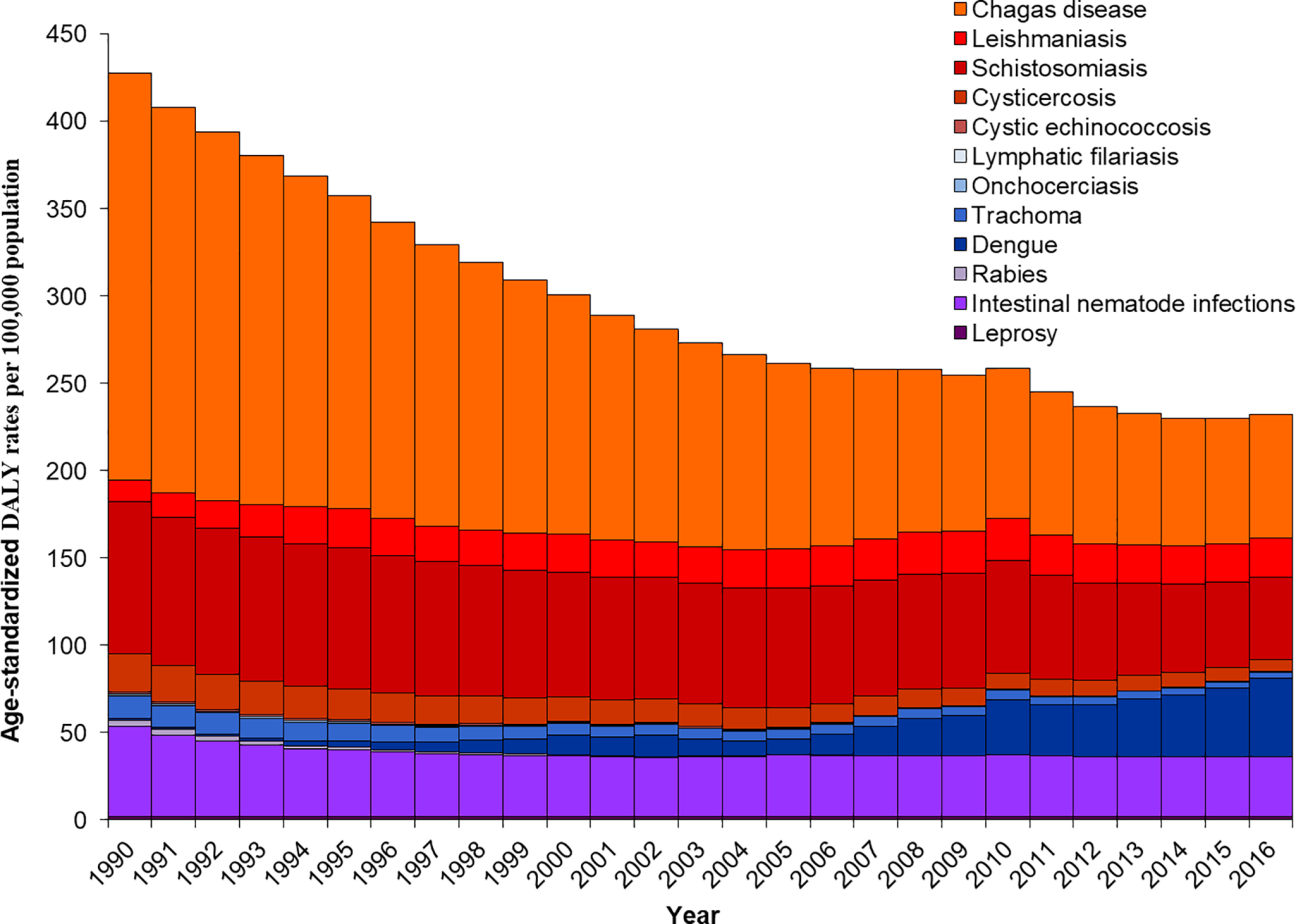
Trends of age-standardized DALY rates (per 100,000 population) from NTD causes in Brazil from 1990 to 2016. DALY = disability-adjusted life-year; NTDs = neglected tropical diseases.

The number of DALYs due to all NTDs combined remained largely unchanged as compared to 1990 (478,686 DALYs; 95% UI: 370,480–645,029), with a small decline of 0.7% over the 27 years (Table 1). By contrast, the age-standardized DALY rates due to all NTDs decreased 45.7% between 1990 and 2016 (Table 1). Fig 3 shows the time trends of age-standardized DALY rates from NTD causes in Brazil from 1990 to 2016. Absolute number of DALYs and age-standardized DALY rates for most NTD causes decreased at national level between 1990 and 2016 (Table 1; Fig 3). The most pronounced decrease in numbers and rates were observed for onchocerciasis, lymphatic filariasis, and rabies (a reduction of 95% or more over the last 27 years). The age-standardized DALY rates due to Chagas disease and ascariasis decreased by 69.7% and 61.7%, respectively. In contrast, both total DALYs and age-standardized DALY rates due to dengue, visceral leishmaniasis, and trichuriasis increased substantially between 1990 and 2016 (Table 1). For some NTD causes such as leprosy, hookworm disease, *cutaneous/mucocutaneous leishmaniasis*, and schistosomiasis, the absolute number of DALYs increased between 1990 and 2016. However, the age-standardized DALY rates for these causes decreased in the period (Table 1).

### Burden by sex and age

In 2016, the national burden of NTDs was slightly higher in males (51.9%; 246,843 DALYs; age-standardized DALY rate of 253.14/100,000 population) than in females (48.1%; 228,567 DALYs; age-standardized DALY rate of 214.35/100,000 population), with a male-female DALY ratio of 1.2. With the exception of cysticercosis and STHs, age-standardized DALY rates for most NTD causes were higher in males.

**Fig 4:**
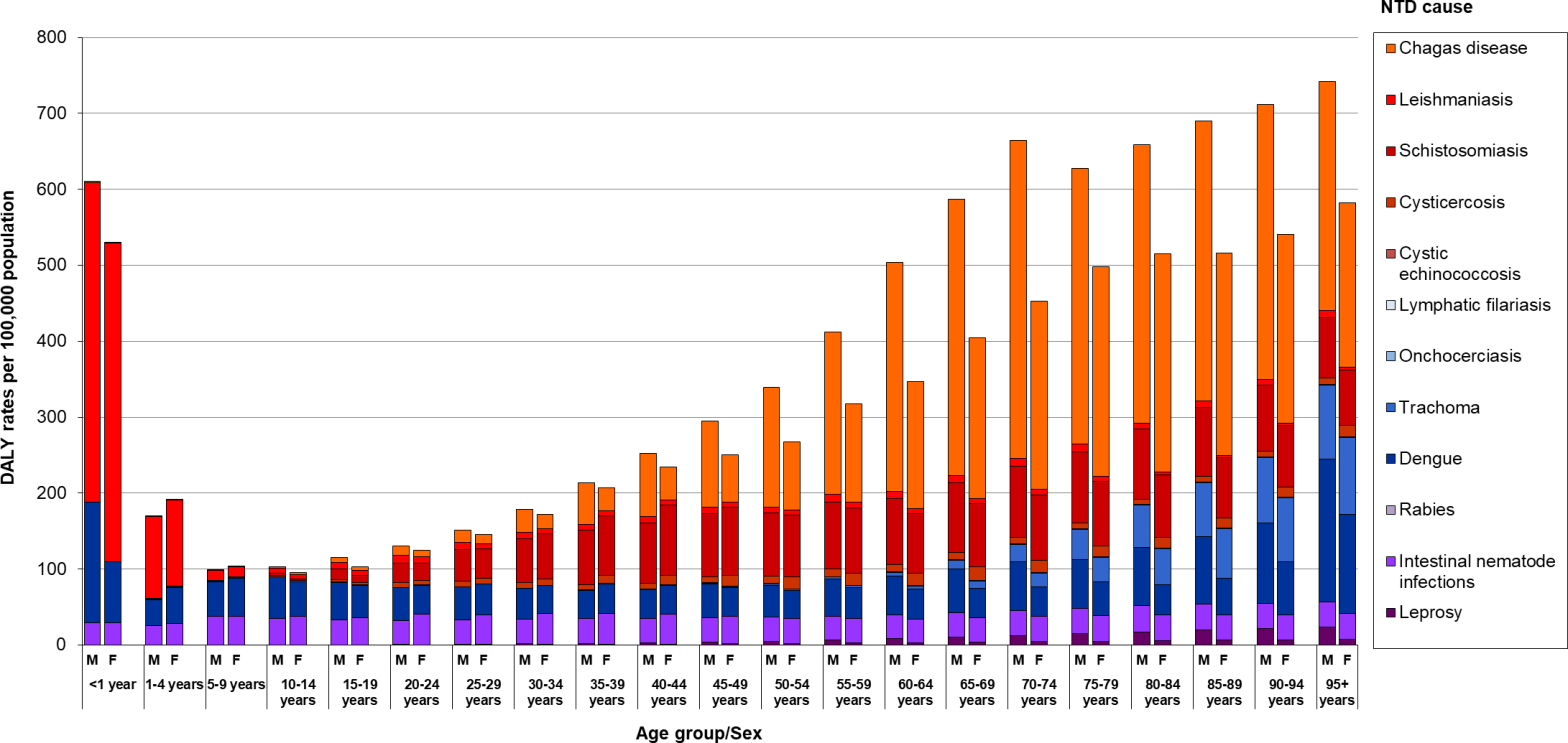
Distribution of DALY rates (per 100,000 population) from NTDs by sex, age group, and cause in Brazil, 2016. DALY = disability-adjusted life-year; NTDs = neglected tropical diseases; M = male; F = female.

In 2016, the burden of NTDs spanned all age groups, with the highest proportion of DALYs in middle-aged adults (aged 35–64 years), with a peak in the aged 55–59 years (S1 Fig). Fig 4 shows the distribution of DALY rates due to NTDs by sex, age group, and cause in Brazil in 2016. DALY rates due to all NTDs were higher for males across all age groups, with exception for age groups 1–9 years. The highest difference of DALY rates between men and women was observed in the age groups older than 50 years (Fig 4). The highest DALY rates (>500 DALYs/100,000 population) for both sexes combined were observed at both extremes of age spectrum (children under 1 year and those aged 70 years and older). DALY rates decreased progressively from the peak of age <1 year to age 10–14 years, and then increased progressively with age and with the peak at age 95+ years (Fig 4; S2 Fig). The age pattern in males and females was somewhat similar to the pattern of both sexes aggregated. However, for males DALY rates also increased progressively from children up to age 70–74 years, decreased at age 75–79 years, and then increased progressively with a peak at age 90+ years (Fig 4).

The age-specific patterns varied between the NTD causes. In childhood, mainly for age 0–4 years, visceral leishmaniasis, dengue, and STHs were the main causes of DALY rates among all NTDs (Fig 4; S2 Fig). After childhood, dengue and STHs were the main causes of DALYs. Schistosomiasis, dengue, Chagas disease, and STHs were the largest causes of DALY rates in young and middle-aged adults. Chagas disease, schistosomiasis, dengue, trachoma, and STHs were the most important causes of DALYs at older age groups (Fig 4; S2 Fig). For the main NTD causes, DALY rates due to leishmaniasis peaked at age <1 year (420.3 DALYs/100,000). Dengue burden peaked at age <1 year (120.9 DALYs/100,000), and age 95 years and older (144.4 DALYs/100,000). STH burden peaked between 5–9 years (37.7 DALYs/100,000). Chagas disease and schistosomiasis peaked between 70–74 years (323.0 DALYs/100,000 and 89.5 DALYs/100,000, respectively). Cysticercosis burden peaked between 65–69 years (14.0 DALYs/100,000). Leprosy and trachoma burden peaked at age 95 years and older (11.4 DALYs/100,000 and 100.4 DALYs/100,000, respectively) (S2 Fig).

### Regional variation in NTD burden

**Fig 5:**
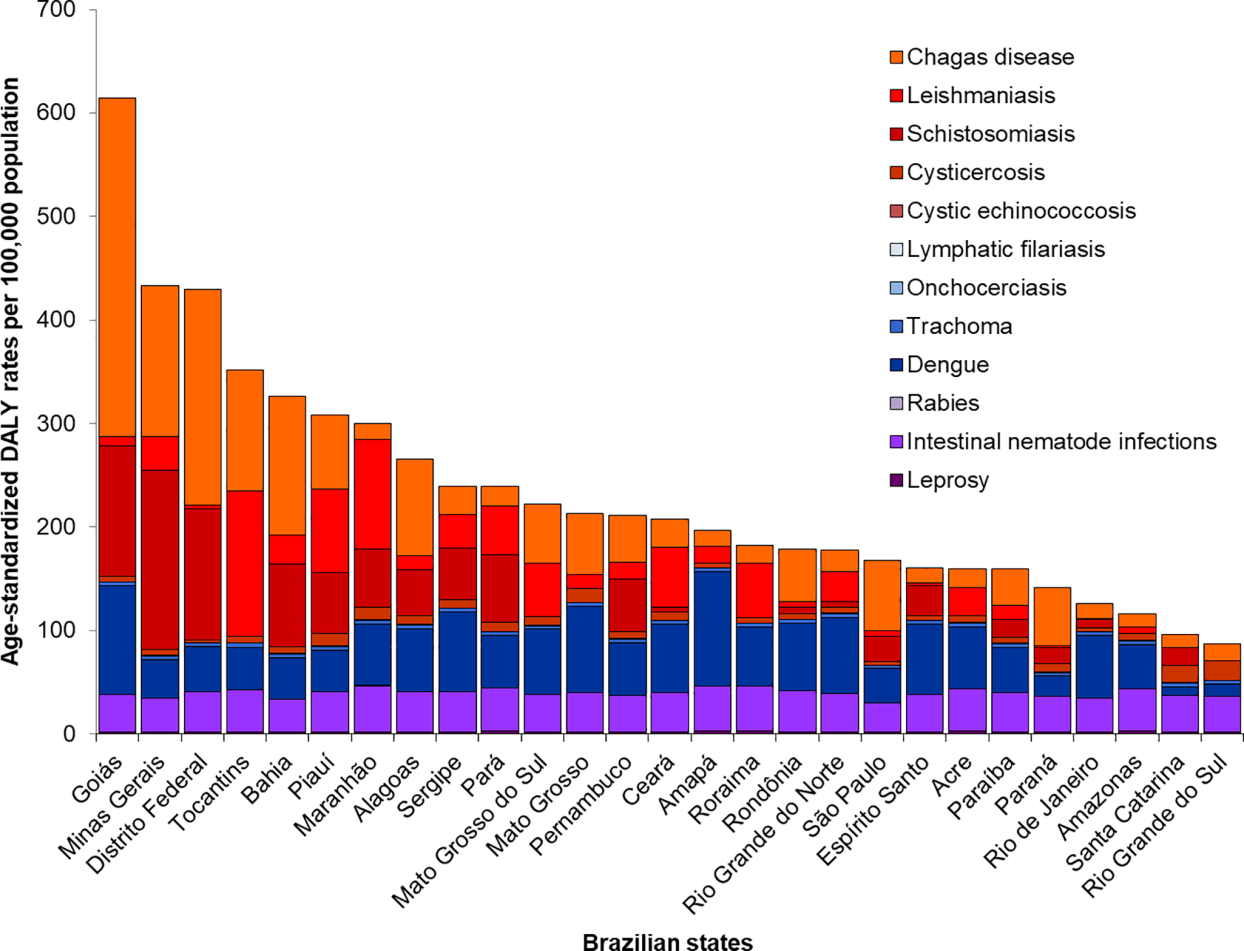
Age-standardized DALY rates (per 100,000 population) from NTDs by states in Brazil, 2016. DALY = disability-adjusted life-year; NTDs = neglected tropical diseases.

There was substantial geographic variation in the burden of NTDs among the Brazilian states. In 2016, the highest age-standardized DALY rates due to all NTDs combined at the state level were observed in Goiás (614.4 DALYs/100,000), Minas Gerais (433.7 DALYs/100,000), and Distrito Federal (430.0 DALYs/100,000) (Fig 5; Table 2). Between 1990 and 2016, the absolute number of DALYs for all NTDs combined presented increase for most Brazilian states, with highest increases mainly in the states of the North and Northeast regions (Table 2). By contrast, age-standardized DALY rates decreased in most Brazilian states, with the greatest declines observed in states with the highest age-standardized DALY rates in 1990, such as Goiás and Distrito Federal (Table 2). The only states with increase in age-standardized DALY rates were Amapá, Ceará, Rio Grande do Norte, and Sergipe (Table 2).

**Table 2:** Absolute number of DALYs and age-standardized DALY rates (per 100,000 population) for all neglected tropical diseases combined by state in Brazil, with percentage change between 1990 and 2016.

Region/State	Number of DALYs (95% UI)			Age-standardized DALY rate (per 100,000) (95% UI)		
	1990	2016	% Change 1990–2016	1990	2016	% Change 1990–2016
***North***						
* *Acre	739.1 (360.1–1,729.3)	1,206.1 (691.7–1,937.7)	63.2	195.66 (102.94–448.29)	159.35 (92.08–257.25)	-18.6
* *Amapá	360.1 (176.5–818.3)	1,459.4 (824.7–2,249.0)	305.2	137.45 (77.44–278.09)	196.35 (112.92–297.07)	42.8
* *Amazonas	4,044.7 (2,454.1–6,540.1)	4,364.5 (2,628.7–6,991.6)	7.9	222.47 (142.28–336.58)	116.41 (71.44–184.20)	-47.7
* *Rondônia	2,280.5 (1,487.4–3,835.6)	2,951.6 (1,926.1–4,332.5)	29.4	314.40 (228.24–473.65)	178.49 (119.63–257.73)	-43.2
* *Roraima	396.6 (225.6–693.1)	924.1 (472.9–1,460.3)	133.0	252.57 (156.93–412.70)	182.12 (98.43–279.77)	-27.9
* *Pará	11,941.3 (6,985.3–21,691.1)	18,958.5 (11,419.1–30,540.9)	58.8	290.00 (174.83–511.93)	239.24 (145.05–384.83)	-17.5
* *Tocantins	2,541.6 (1,661.8–4,058.7)	5,142.9 (3,382.2–7,569.2)	102.3	389.58 (290.23–547.43)	351.99 (238.77–507.74)	-9.6
***Northeast***						
* *Alagoas	8,123.1 (5,569.2–12,315.6)	8,306.1 (5,694.8–12,132.5)	2.3	436.09 (319.48–618.87)	265.57 (185.12–382.65)	-39.1
* *Bahia	43,843.8 (31,849.8–66,129.9)	48,438.6 (33,810.1–70,274.6)	10.5	509.31 (389.98–705.91)	326.52 (230.32–470.52)	-35.9
* *Ceará	10,772.3 (6,462.9–19,073.8)	18,084.2 (11,483.5–27,761.0)	67.9	179.71 (115.33–294.57)	207.55 (131.78–317.25)	15.5
* *Maranhão	15,323.1 (8,852.5–27,309.0)	20,563.5 (12,334.2–33,248.2)	34.2	300.43 (182.21–509.73)	299.84 (181.50–481.34)	-0.2
* *Paraíba	5,324.8 (3,312.0–8,823.2)	6,199.2 (4,026.9–9,252.0)	16.4	192.32 (130.01–295.11)	159.35 (103.18–237.18)	-17.1
* *Pernambuco	22,479.5 (14,926.2–36,347.9)	19,534.9 (13,024.6–29,186.2)	-13.1	392.18 (271.47–599.88)	211.10 (142.23–313.37)	-46.2
* *Piauí	7,580.0 (4,592.5–12,629.6)	9,423.0 (5,917.2–14,842.6)	24.3	379.43 (247.38–592.36)	307.90 (195.34–481.88)	-18.9
* *Rio Grande do Norte	3,571.7 (2,059.8–6,290.8)	5,983.0 (3,705.4–9,000.7)	67.5	161.36 (101.17–261.00)	177.61 (110.51–269.52)	10.1
* *Sergipe	2,986.3 (1,767.4–5,054.8)	5,159.8 (3,157.3–8,077.6)	72.8	234.98 (152.23–367.65)	239.48 (146.93–374.05)	1.9
***Southeast***						
* *Espírito Santo	3,862.5 (2,233.0–6,913.2)	6,465.8 (4,074.9–9,893.7)	67.4	176.69 (109.07–298.44)	160.53 (101.24–245.80)	-9.1
* *Minas Gerais	114,331.2 (87,474.0–156,433.9)	94,902.5 (63,384.4–145,372.8)	-17.0	1,015.81 (804.16–1,335.20)	433.66 (290.50–663.04)	-57.3
* *Rio de Janeiro	15,751.6 (10,051.1–25,977.1)	21,835.9 (14,032.2–32,795.6)	38.6	136.19 (89.70–215.29)	126.55 (81.13–190.07)	-7.1
* *São Paulo	97,614.7 (75,179.6–134,028.3)	77,800.1 (55,786.7–109,574.0)	-20.3	392.04 (311.94–518.78)	167.64 (120.22–236.35)	-57.2
***South***						
* *Paraná	20,577.6 (15,399.6–30,272.1)	16,387.2 (11,216.9–23,908.3)	-20.4	335.84 (265.47–453.00)	141.52 (97.10–205.59)	-57.9
* *Rio Grande do Sul	11,933.9 (7,147.7–20,218.3)	10,688.5 (5,784.3–17,980.7)	-10.4	149.00 (93.31–241.31)	87.20 (46.65–146.92)	-41.5
* *Santa Catarina	6,602.3 (3,721.2–11,698.4)	6,853.4 (3,750.2–11,789.8)	3.7	178.15 (106.94–296.97)	96.48 (53.26–164.29)	-45.8
***Central-West***						
* *Distrito Federal	11,651.4 (8,921.6–16,088.4)	12,275.7 (8,423.3–18,162.6)	5.4	1,202.69 (976.45–1547.75)	430.00 (300.99–626.44)	-64.2
* *Goiás	45,993.9 (37,873.8–58,524.2)	38,804.1 (27,990.4–54,059.8)	-15.6	1,940.90 (1,655.07–2,351.63)	614.35 (453.35–841.49)	-68.3
* *Mato Grosso	4,041.3 (2,550.5–6,933.6)	6,811.0 (4,504.8–9,819.5)	68.5	281.72 (197.35–432.50)	212.67 (142.06–303.45)	-24.5
* *Mato Grosso do Sul	4,016.9 (2,772.7–6,162.6)	5,886.3 (3,891.8–8,533.9)	46.5	300.31 (227.56–412.08)	222.55 (148.56–320.05)	-25.9

DALYs = disability-adjusted life-years. 95% UIs = 95% uncertainty intervals.

**Fig 6:**
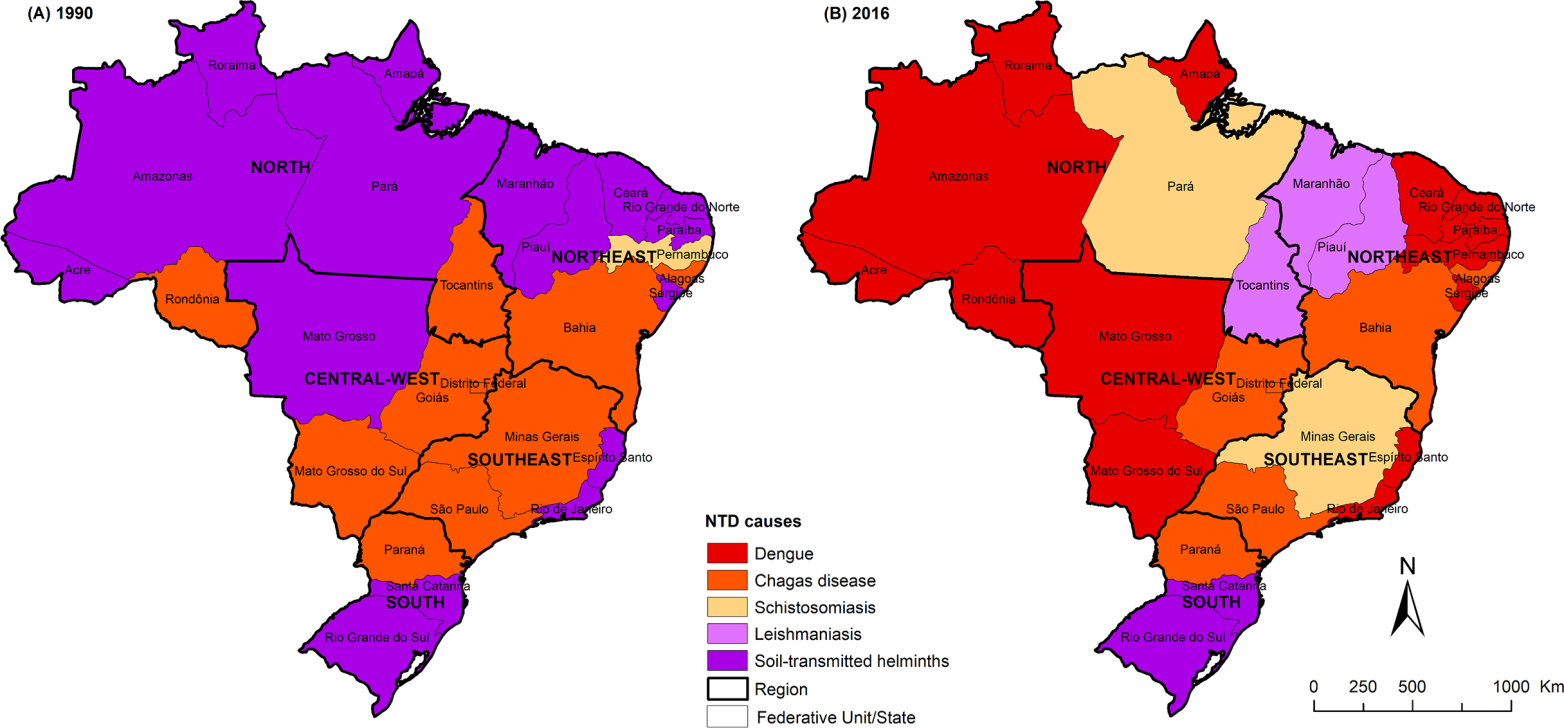
Spatial distribution of leading causes of total DALYs among all NTDs by state in Brazil for (A) 1990 and (B) 2016. DALYs = disability-adjusted life-years; NTDs = neglected tropical diseases. Choropleth map produced using ArcGIS version 9.3 (Esri, Redlands, CA, USA). Source of shapefile: Brazilian Institute of Geography and Statistics (IBGE in Portuguese; https://mapas.ibge.gov.br/bases-e-referenciais/bases-cartograficas/malhas-digitais.html).

Fig 6 shows the main causes of total DALYs among NTDs by Brazilian state in 1990 and 2016. There was a temporal variation among the main causes of NTDs by Brazilian states. In 1990, STHs was the leading cause of total DALYs among NTDs in 16 states and Chagas disease in 11 states. In 2016, dengue was the leading cause in 14 Brazilian states and Chagas disease in six states, while STHs ranked first only in two states (Fig 6).

**Fig 7:**
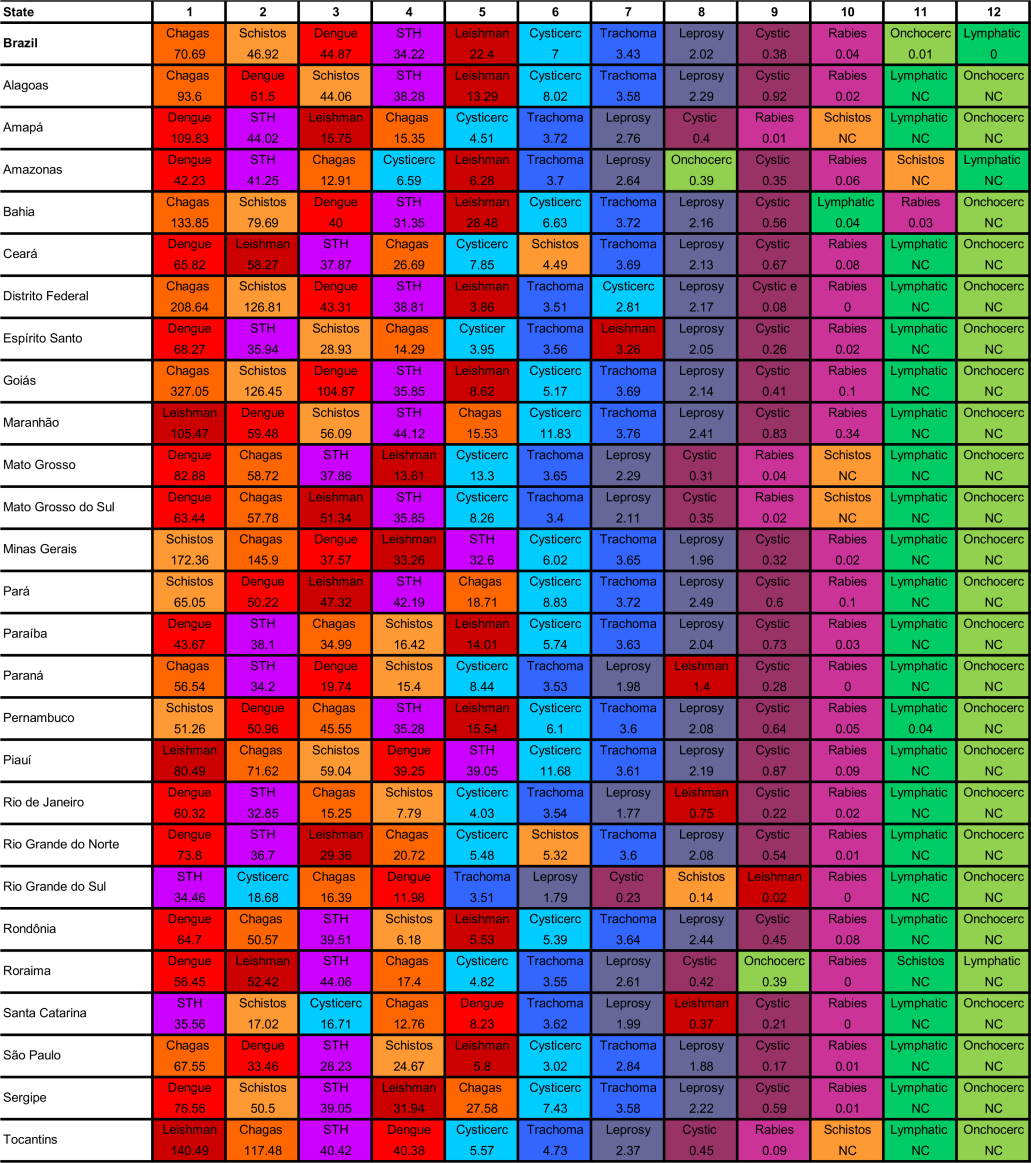
Ranking of age-standardized DALY rates (per 100,000 population) for NTD causes by state in Brazil, 2016. NC = not calculated due to lack of available epidemiological data or non-endemic area. Chagas = Chagas disease; Schistos = Schistosomiasis; STH = Soil-transmitted helminths/Intestinal nematode infections; Leishman = Leishmaniasis; Cysticerc = Cysticercosis; Cystic = Cystic echinococcosis; Onchocerc = Onchocerciasis; Lymphatic = Lymphatic filariasis; DALY = disability-adjusted life-year; NTD = neglected tropical disease.

Fig 7 shows the ranking of age-standardized DALY rates of the specific NTD causes by Brazilian state in 2016. Among the leading causes of NTD burden at the national level, dengue, Chagas disease, and STHs ranked among the top five NTD causes in all 27 Brazilian states. Schistosomiasis was among the top leading five NTD causes in 17 of 27 Brazilian states and leishmaniasis, in 22 states (Fig 7).

## Discussion

To the best of our knowledge, this is the first comprehensive overview of the epidemiological situation and trends regarding the NTD burden in Brazil. GBD 2016 results showed a consistent reduction of age-standardized DALY rates for all NTDs combined at national level from 1990 to 2016. Chagas disease was the main cause of DALYs, with a decreasing trend in the period. The disease burden was higher among males, in the youngest and the eldest, and endemic states for the major NTDs (Chagas disease, schistosomiasis, and dengue). Despite the decline in DALY rates between 1990 and 2016, the absolute number of DALYs due to NTDs remained virtually unchanged, evidencing NTDs as important cause of disability and premature death in Brazil.

### NTD burden by sex and age group

Our findings clearly show that NTDs are important causes of health loss for both men and women, but there was considerable sex difference in the burden of many NTDs. Age-standardized DALY rates for all NTDs combined and most causes were higher in males as compared to females, reflecting the patterns for most of these diseases observed in other Brazilian large-scale epidemiological studies [10,34–39]. Despite this pattern, the relationship between gender and infection risk is controversial, and the causes of higher male susceptibility for some NTDs is still a matter of debate [37]. The observed findings indicate gender-specific patterns of infectious disease exposure, as the relationship between gender and risk of infection is often conditioned by different socioeconomic, environmental, occupational, and behavioral factors, as well as access to healthcare services [10,36,37,40]. In fact, healthcare seeking behavior in Brazilian males is more often retarded, with increased morbidity and severity for some diseases [39,41].

The high NTD burden in children under 1 year was mainly due to the high impact of premature mortality caused by visceral leishmaniasis, confirming the well-known pattern of disease occurrence in the child population [42,43]. The high burden from dengue in childhood is mainly due to the increase in severe and fatal cases of the disease among children in recent years, related to the simultaneous circulation of different serotypes in several areas of the country [26,44,45]. In addition, the high burden of NTDs in more advanced age groups can be explained by the chronicity nature of major NTDs with high mortality and morbidity impact, such as Chagas disease, schistosomiasis, leprosy, trachoma, and cysticercosis [10,35,37,41,46]. The higher burden of dengue in the elderly may reflect the simultaneous occurrence of common chronic comorbidities, such as cardiovascular diseases and cancers, increasing complications, severity, and case fatality of NTDs in this age group [41,47,48]. The elderly population should receive special attention from the moment of clinical suspicion to diagnosis and treatment of these diseases [34,40,47]. Furthermore, the considerable DALY rates among younger and economically productive age groups call for further improvements in disease-specific control actions in areas with high transmission [35,36,48].

### Burden by specific NTDs

Chagas disease is responsible for most NTD deaths recorded in the country [10]. The high burden due to Chagas disease corroborates findings of previous large-scale studies in Brazil, using mortality data [10,11,39]. Other major NTDs with predominantly chronic evolution were important causes of disability and/or premature death, such as schistosomiasis, leishmaniasis, cysticercosis, STHs, and trachoma [36,37,41,42,49–51]. For some chronic NTDs, such as Chagas disease, the highest disease burden is a result of infection in previous years. These data reinforce the need to improve epidemiological surveillance, and clinical management and to ensure adequate access to the healthcare system (diagnosis, treatment, management, and follow-up of cases) and social support for individuals affected by these diseases [11,36,37,39,52].

STHs are important causes of disease burden throughout the national territory, mainly among the most underprivileged population groups [49,53]. Dengue fever is ranked as the third leading NTD cause in 2016. Dengue is currently the NTD with the highest absolute number of new cases in Brazil, with a relatively low case fatality rate [26,45]. The disease has a wide geographical distribution in the country and, despite the intensification of control measures, there has been an increase in the number of severe cases, hospitalizations, and deaths in recent years [26,40,44,45]. This pattern is reflected by the highest proportion of YLLs in relation to total DALYs for dengue in 2016.

### Changes over time of NTD burden

The steady decline at national level between 1990 and 2016 of age-standardized DALY rates for all NTDs combined may be caused mainly by the decline of the burden for the main NTDs, such as Chagas disease and schistosomiasis. These findings corroborate the observed patterns and trends for the main NTDs in recent years, and can be attributed mainly to the impact of the specific-disease surveillance and control programs implemented in the last decades [11,36,37,52,54–56]. For Chagas disease, the implementation of control measures for vector and blood-borne transmission–such as systematic entomological surveillance and screening of blood donors–reduced considerably the number of new cases and deaths in the last decades [37,39,52,54,57]. A more pronounced decline of absolute numbers and rates of DALYs due to NTDs was observed in the highly endemic states for Chagas disease in the past, such as Goiás, São Paulo, and Minas Gerais [57,58]. With the control of the vector domiciliary transmission of Chagas disease by its principal vector, the kissing bug *Triatoma infestans*, other types of transmission have become more relevant. These are directly related to the enzootic cycle of infection, such as extra-domiciliary vector transmission and domiciliary without vector colonization, and oral transmission [52,59]. In fact, oral transmission was the most frequent infection route of acute cases recorded in Brazil in recent years, mostly in the Amazon region [52]. However, due to its chronic nature, the challenge of recognizing chronic Chagas disease in the health services network through surveillance actions persists in Brazil [39].

Due to impact of surveillance and control program measures based mainly on large-scale treatment of risk populations in endemic areas, morbidity and mortality of schistosomiasis has been reduced, mainly in Northeast Brazil [36,48,55]. However, the wide geographical distribution of intermediate snail hosts, internal migration, tourism activities, and poor sanitary conditions still favor the persistence and expansion of disease foci [36,55].

There was a drastic reduction in human rabies cases in Brazil during the last decades, mainly due to systematic prevention and control activities directed to the control of urban canine rabies and post-exposure prophylaxis after aggression by suspect animals [56]. However, there are still endemic areas in which the urban cycle prevails, especially in the Northeast region [56,60]. At the same time, there has been observed an emergence and expansion of sylvatic transmission cycle, with increasing importance of blood-feeding bats in Brazil [56,60,61]. This highlights the need for improvement and maintenance of surveillance and control of rabies aimed at the urban cycle and its implementation in the sylvatic cycle [56].

Other factors not related to disease-specific control programs, in particular for NTDs without systematic surveillance and control programs or compulsory notification, such as cysticercosis and STHs, may have played an important role in the decline of burden for some NTDs in Brazil, such as improvements in socio-economic and sanitary conditions, increased urbanization, improved health education and access to healthcare services [8,9,11,41,49].

In contrast, the age-standardized DALY rates due to dengue and visceral leishmaniasis showed consistent increase. In fact, despite of the efforts of specific control programs, the measures to reduce transmission of these diseases has not proven to be sufficiently effective [40,62,63], and the failures in the control of these infectious diseases favored the increase of morbidity and mortality in recent years [26,40,42,44]. There has been an increase in mortality and case fatality from visceral leishmaniasis in the last decades, related to the introduction of the disease in new geographic areas and unfavorable host factors, such as malnutrition, immunosuppression (mainly due to HIV coinfection), and other comorbidities [42,64]. The large increase of dengue burden in Brazil is consistent with the wide geographical spread of the mosquito vector, and simultaneous circulation of multiple dengue virus serotypes [26,40,44].

Despite of the decrease in the age-standardized DALY rates for all NTDs combined between 1990 and 2016, the absolute number of DALYs remained practically the same in the period. A particular trend has been observed for some causes such as leprosy and hookworm disease, with an increase in the number of DALYs, but with a decrease in age-standardized rates during the period. This pattern may suggests that the increase in absolute numbers is mainly attributable to demographic changes such as population growth and changes in population age structure [12].

GBD 2016 findings also show that the observed burden for the main NTDs, was higher than expected for the country based on SDI. This implies that the income per capita, educational levels and fertility rates were not commensurate with the high burden for some NTDs in Brazil, and despite the decrease in DALY rates, the impact for some diseases are much higher than expected for the socioeconomic development status of the country [65]. In addition, the current political and economic crisis in the country has widened poverty and social inequalities. This has potential significant negative impacts on policies and actions for health care and surveillance, as well as education and research. Together, they can increase the burden of NTDs in Brazil in the future.

### Geographical differences in Brazilian states

There was a substantial geographic variation in NTD burden in Brazil, with occurrence of health lost due to NTDs in all 27 Brazilian states. The observed geographic differences in NTD burden in Brazil are due to geographical distribution of human prevalence and incidence, vectors and/or reservoirs associated with each disease, as well as socioeconomic, demographic and environmental conditions, sanitation, quality of health surveillance, and access to healthcare services for diagnosis and treatment. These factors favor the maintenance, transmission and spread of these diseases, with consequently negative impact on morbidity, disability, and premature mortality [8,9,11].

In 2016, with the exception of Minas Gerais (Southeast region), the highest age-standardized DALY rates were observed in the states of the Central-West, North, and Northeast regions. This observed pattern reflects mainly the presence of areas highly endemic for important NTDs in the past and present, especially for Chagas disease (Central-West and Southeast regions), schistosomiasis (Northeast and Southeast regions), and leishmaniasis (Northeast and North regions) [11,36,39,42,58]. In 2016, dengue was the predominant cause of NTD burden in 13 states located mainly in the North and Northeast regions, reflecting a marked increase of incidence, number of severe forms, and deaths from dengue, contributing to the increase in the loss of healthy years of life due to disease in recent years [26,45]. The states of the South region, the most socioeconomically developed region, showed the lowest age-standardized DALY rates due to NTDs in 2016 [8,11].

### Surveillance, prevention and control initiatives for NTDs and health policy implications

Currently, there is a global and regional effort directed to face the NTDs [2,66–68]. The launching of the WHO NTD roadmap and the London Declaration on NTDs contributed significantly to these global efforts [66–68]. In 2015, NTDs have been included in the Sustainable Development Goals (SDGs), with the goal 3.3 to end the epidemics of AIDS, tuberculosis, malaria, and NTDs and combat hepatitis, water-borne diseases, and other communicable diseases by 2030 [39,69]. In line with global initiatives, Brazil launched in 2012 an integrated plan of strategic action to eliminate some important NTDs, such as leprosy, filariasis, schistosomiasis and onchocerciasis as a public health problem, trachoma as an important cause of blindness, and more effective control of STHs [70]. In conjunction with states and municipalities, local plans for the elimination of these diseases should be developed throughout Brazil, promoting public health and social inclusion actions, consistent with the principles of the Brazilian Unified Health System (*Sistema Único de Saúde—SUS*) [39,70]. In 2016–2017, Brazil has completed the fourth edition of this national annual campaign–about 6 million school-aged children (5–14 years) had been screened in public schools of Brazilian municipalities with higher social vulnerability and high disease risk for leprosy, trachoma, and schistosomiasis, with treatment of positive cases (including household contacts), and received preventive chemotherapy against STHs [71].

For effective and sustainable control of NTDs, specific control measures should be developed in conjunction with other integrated inter-sectoral public policies, such as human rights, improvements in social conditions, access to adequate water and sanitation, improved access to health care services and health education [9,11,36,39,48,72–74]. A higher priority should be given to research and expansion and improvement of health technologies (drugs, vaccines, diagnostics, and control methods) for NTDs [72]. Both financial and technical management will have to be decentralized even more to state and municipal governments, for structuring the capacity to implement interventions for surveillance, control and prevention of cases and deaths by NTDs in endemic areas [11,48,70]. There is a clear need to integrate care and attention to these conditions into the network of health care in the *SUS* with a high priority to primary health care [35,39]. Integrated access and quality of health care should also be guaranteed for the diagnosis and management of chronic comorbidities (e.g. hypertension and diabetes mellitus) and coinfections (e.g. HIV/AIDS), since the presence of these NTD patients could aggravate the disease evolution, increasing the morbidity and mortality [26,47,75]. In addition, the implementation and sustainability of appropriate surveillance and control mechanisms and a mandatory reporting system for some important NTDs, such as cysticercosis, STHs and chronic Chagas disease, throughout the national territory could provide more accurate epidemiological data on the population prevalence and would allow geographical mapping of the affected areas [39,41,49]. In fact, the knowledge of true NTD burden is essential to track health progress, assess the impact of public health interventions, and inform evidence-based policy decisions [16].

### Limitations

Overall limitations of GBD 2016 study have been published in detail elsewhere [12–14,22]. Some specific limitations related to NTDs estimated in GBD studies were described in previous publications, such as coverage, quality, and availability of epidemiological data used to estimate the disease burden [5,16].

Despite considerable improvements of quality since the 1990s, mortality data differ in coverage and quality among Brazilian states, which may have cause underestimations especially in the Northeast region [10,37,46,76]. The GBD study used comparable and standardized methods for correction of underreporting of deaths and redistribution of garbage codes [13]. In addition, because of the lag time between mortality data reporting and the availability of databases, estimates for 2016 are mainly based on data and trends from recent years [77].

For non-fatal estimation, epidemiological data available for some NTDs, especially for diseases without national surveillance and control programs and/or mandatory reporting system such as cysticercosis, are scarce [41,78]. When data are of poor quality or unavailable for a location (subnational unit, country, or region), GBD estimates are based on model covariates and data available from neighboring locations with a similar health profile, which may be less precise [14,15,30,79].

ICD coding rules allow only a single underlying cause of death and the GBD assumes that some NTD causes (e.g. cutaneous leishmaniasis and leprosy) have no mortality or are considered a rare event (total DALYs for theses causes are equal to the total YLDs), possibly leading to underestimation [13,16,32,80]. The underlying causes of death may have been coded as a complication or aggravation associated with some NTDs (such as gastrointestinal bleeding, portal hypertension, and esophageal varices for schistosomiasis, and heart failure for Chagas disease) [10,11,16,36,47,48]. For some NTDs in which death is considered rare or with few records, the non-inclusion of these in the YLL calculation may substantially underestimate the total DALYs in higher endemic locations.

GBD estimates are intended to only capture the direct health loss due to a specific cause in an individual [5,81]. They do not consider the social and economic impact and stigmatization of NTDs in the affected individuals, their families and communities [5,32,33,81]. Thus, the estimates of disease burden are partial measures of the impact and consequence of NTDs for the society [81].

### Conclusions

NTDs continue being an important cause of disability and premature death in Brazil, since most diseases are preventable and/or treatable with highly efficient interventions. NTDs contribute considerably to the loss of health in individuals of all ages in all Brazilian states, with a higher burden among males, youngest (children under 1 year) and oldest age groups (aged 70 years and older).

Our findings call for renewed and comprehensive efforts to control and prevent the NTD burden in Brazil through evidence-based interventions. Integrated control and surveillance measures should focus on vulnerable population groups and geographic areas with highest NTD burden.

## Supporting information

S1 Fig Distribution of absolute number of DALYs due to NTDs by sex, age group, and cause in Brazil, 2016. DALYs = disability-adjusted life-years; NTDs = neglected tropical diseases.
(TIF)

S2 Fig DALY rates (per 100,000 population) from NTDs by age group in Brazil, 2016. DALYs = disability-adjusted life-year; NTDs = neglected tropical diseases.
(TIF)
