# Supplementary figures and images for: The burden of Neglected Tropical Diseases in Brazil, 1990-2016: A subnational analysis from the Global Burden of Disease Study 2016
Source: PLoS Negl Trop Dis. 2018 Jun 4;12(6):e0006559. doi: 10.1371/journal.pntd.0006559 (PMC6013251; doi:10.1371/journal.pntd.0006559)

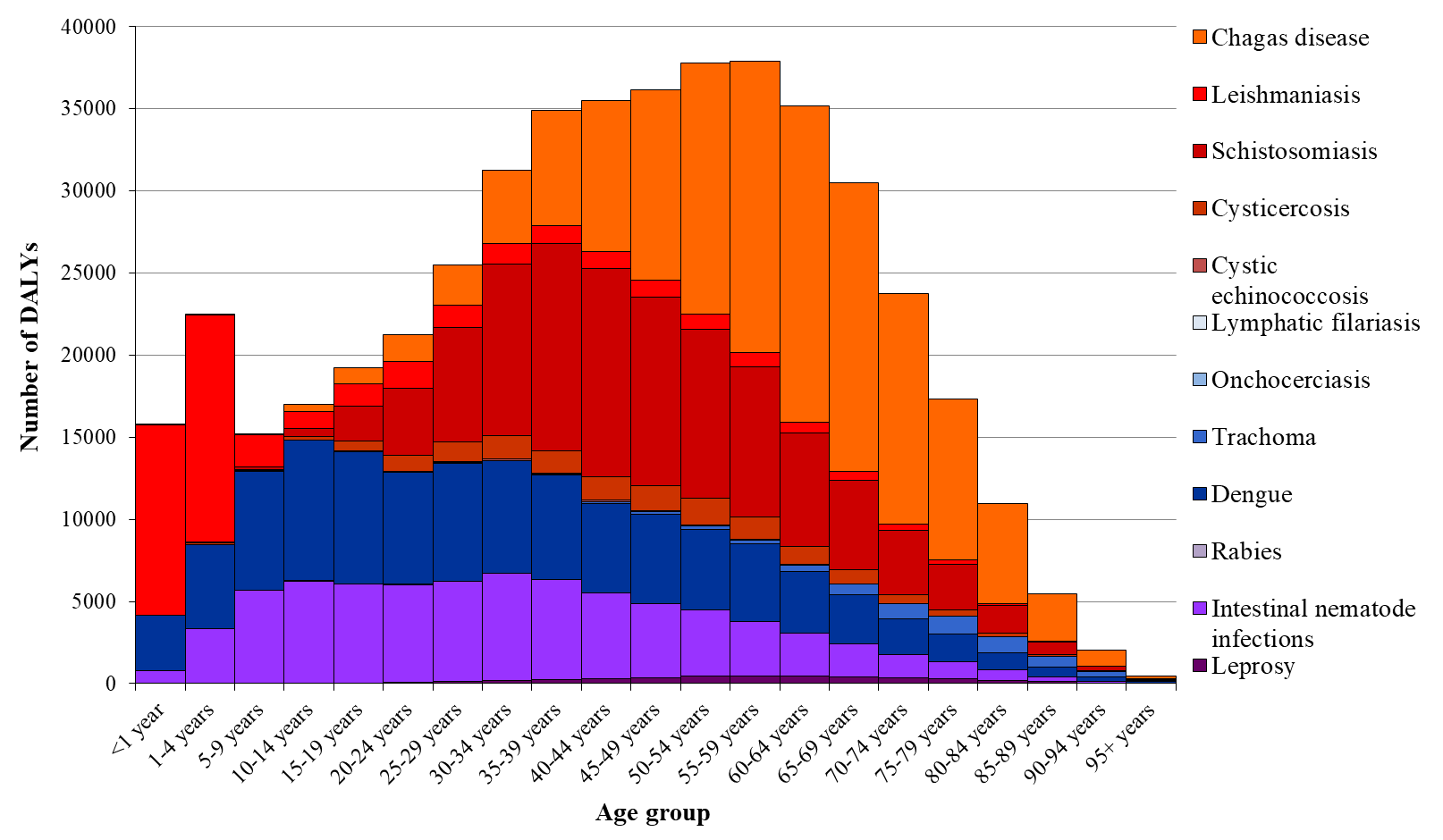

Supplement: S1 Fig — DALYs = disability-adjusted life-years; NTDs = neglected tropical diseases. (TIF) [file pntd.0006559.s001.tif]

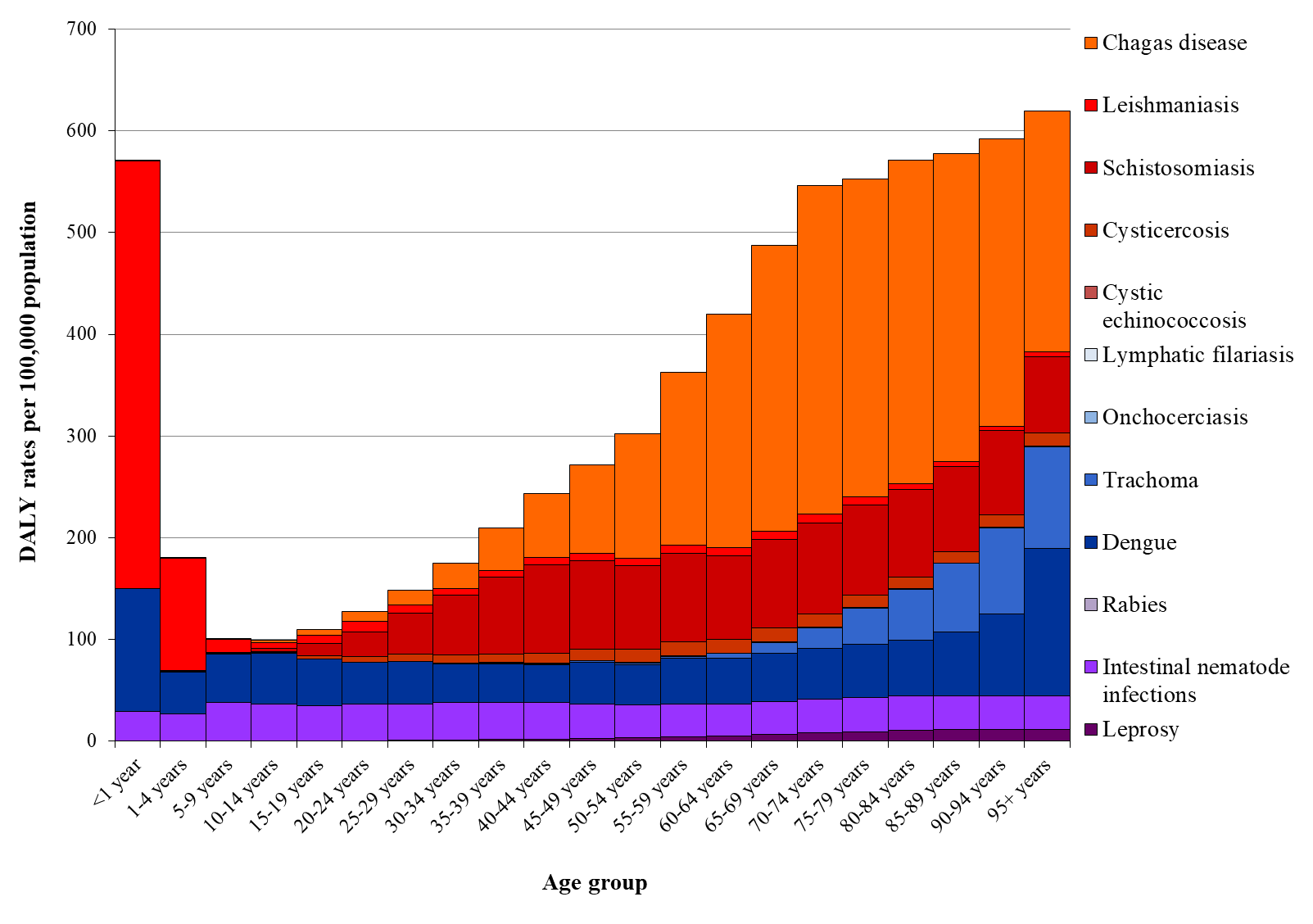

Supplement: S2 Fig — DALYs = disability-adjusted life-year; NTDs = neglected tropical diseases. (TIF) [file pntd.0006559.s002.tif]
